# Modern Therapeutic Strategies in Endodontics and Restorative Dentistry

**DOI:** 10.3390/medicina59020333

**Published:** 2023-02-10

**Authors:** Alfredo Iandolo

**Affiliations:** Department of Endodontics, University of Dentistry, 84121 Salerno, Italy; aiandolo@unisa.it

Modern endodontics aims to reduce or eliminate bacterial load inside root canals. Moreover, this goal can be achieved through correct shaping, 3D cleaning, and 3D obturation of the complex root canal system. Consequently, endodontically treated tooth restoration occurs [1,2,3].

On the other hand, for teeth with complex anatomies or immature apices and teeth that have already been treated endodontically but incorrectly, their treatment can be more challenging.

Currently, with advances in modern technologies, the above-mentioned difficult cases can be treated safely and reliably. In the diagnostic phase, it is now possible to analyze details, which cannot be seen with traditional radiography, with the help of 3D radiography and CBCT. Consequently, a proper treatment plan can be carried out correctly and with greater precision [4,5,6]. Modern technologies, such as microscope and ultrasonics, are very useful during the access cavity phase, especially in cases with previous endodontic treatment.

In addition to access cavity, the other three fundamental phases for obtaining success in endodontics are shaping, 3D cleaning, and 3D obturation.

The shaping phase involves rotating files to widen the root canals mechanically. Presently, with the introduction of new martensitic alloys, even less experienced operators can safely shape complex canals, such as those with severe curvatures [7,8,9].

Immediately after the shaping phase is over, it is important to use a powerful 3D cleaning protocol. Success in endodontics is achieved by eliminating or reducing the bacterial load within a complex endodontic space. Hence, most of the lateral anatomies are unreachable by rotary files, such as lateral canals, loops, isthmuses, and dentinal tubules. Thus, to continue lowering the microbial load, we must use irrigants. However, chemical disinfection activation should be applied to take advantage of its cleaning effect. Three-dimensional cleaning activation techniques include sonic, ultrasonic, heat, and laser activation [10,11,12,13]. Finally, all shaped and cleaned spaces must be filled with bioactive materials after proper cleansing. Therefore, the three-dimensional filling phase is next, where the gutta-percha is compacted together with the latest generation of sealers [14,15,16]. Once the endodontic treatment is completed, the tooth must receive a final restoration to avoid coronal bacterial leakage or fractures under masticatory loads.

This Special Issue aims to showcase recent research studies on modern endodontics. Full papers of original studies, short communications, and review articles are invited.
